# *Zophobas morio* larvae as a novel model for the study of *Acinetobacter* virulence and antimicrobial resistance

**DOI:** 10.3389/fmicb.2024.1375787

**Published:** 2024-02-27

**Authors:** Nadya Rakovitsky, Elizabeth Temkin, Amichay Hameir, Mor Lurie-Weinberger, Alona Keren-Paz, Yehuda Carmeli

**Affiliations:** ^1^National Institute for Antibiotic Resistance and Infection Control, Israel Ministry of Health, Tel Aviv, Israel; ^2^School of Medicine, Tel Aviv University, Tel Aviv, Israel

**Keywords:** *in vivo* model, *Acinetobacter baumannii*, *Zophobas morio*, larvae, antibiotic-resistant bacteria

## Abstract

The use of mammalian models for *in vivo* testing of bacterial virulence raises ethical concerns and is expensive and time-consuming. As an alternative, non-mammalian models are sought. *Galleria mellonella* larvae have been used as a model to study several bacterial pathogens. However, their maintenance is challenging, and commercial supply is low. In this study, we aimed to establish the *Zophobas morio* larvae as an alternative non-mammalian model for the evaluation of the pathogenicity and antimicrobial susceptibility of *Acinetobacter baumannii*. We infected *Z. morio* with *Acinetobacter* strains and determined the optimal temperature and inoculum. To visualize the bacterial distribution within the larvae, hematoxylin and eosin (H&E) staining was performed. Next, a survival model of infected larvae was established, and virulence was compared between strains. The effect of antimicrobial treatment in relation to antibiotic susceptibility was studied. Our results demonstrate that *Z. morio* can be used as a model system for *in vivo* studies of *A. baumannii*.

## Introduction

The availability of *in vivo* infection models is of critical importance to infectious disease research (Rong and Liu, 2023). Historically, rodent models served as the primary experimental platform for studying host–pathogen interactions and virulence and for evaluating potential therapeutics due to their genetic, physiological, and immunological similarities to humans. However, the use of rodents raises significant ethical concerns related to animal welfare. To address these concerns, researchers are urged to prioritize humane practices, minimizing pain, distress, and discomfort. Ethical guidelines highlight the importance of “replacement,” advocating for alternative models, such as invertebrate and *in vitro* systems, to reduce reliance on rodent models.

Apart from ethical concerns, rodent models are often expensive, logistically demanding, and time-consuming. Hence, the exploration of alternative models is crucial for preclinical research. Invertebrate models, such as *Caenorhabditis elegans* (nematodes) (Sifri et al., 2005) and *Galleria mellonella* (wax moth larvae) (Meir et al., 2018), are gaining prominence in bacterial infection research (Mylonakis et al., 2005; Seed and Dennis, 2008; Meir et al., 2018; Wang et al., 2023). These models offer genetic tractability, short life cycles, and cost-effectiveness (Supplementary Table S1). Despite their evolutionary distance from mammals, these insect models possess an innate immune system analogous to that of higher organisms. While invertebrate models lack certain physiological aspects of mammals, they significantly contribute to understanding fundamental aspects of host defense and pathogen virulence.

Invertebrates present an ethically acceptable alternative due to their lack of higher-order cognitive functions and complex nervous systems. They are relatively inexpensive to maintain, requiring minimal resources compared to traditional rodent models. The model’s simplicity enables conducting high-throughput experiments (Kerr et al., 2022), facilitating the screening of multiple bacterial strains, in a time-efficient manner.

*G. mellonella* serves as a valuable model for initial screenings and investigations not requiring the more complex mammalian systems. Its relatively large size allows the injection of drugs to study antibiotic effectiveness. Studies using *G. mellonella* have demonstrated a good correlation with results obtained from mammalian models, particularly in evaluating virulence factors, assessing antimicrobial efficacy, and understanding the basic principles of bacterial pathogenesis (Peleg et al., 2009; Loh et al., 2013; Wand et al., 2013; ten et al., 2023; Wang et al., 2023).

However, challenges exist in obtaining *G. mellonella* larvae from commercial suppliers. Only a few countries produce *G. mellonella* for research use, while in many others, there is limited access to a commercial supply of research-grade larvae. Suppliers may differ in the quality of larvae and the conditions of rearing, with some employing antibiotics to obtain research-grade larvae. Limited availability of high-quality larvae, combined with challenges in shipping live animals across borders, poses logistical difficulties. *G. mellonella* larvae typically survive for 5–6 weeks under ideal conditions, but their lifespan can be affected by factors such as temperature, humidity, and food availability. The shipping process becomes critical as delays can shorten the narrow time window available for experimentation. Reproducing and maintaining *G. mellonella* in-house is labor-intensive, increases the costs of the experiments, and is not suited to large-scale experiments.

In this study, we aimed to develop an alternative invertebrate infection model for studying the virulence of *Acinetobacter baumannii* and to examine the effectiveness of antimicrobials in this model. For our study, we selected the insect host *Zophobas morio* (Coleoptera: *Tenebrionidae*), which is a darkling beetle species commonly referred to as the “superworm.” *Z. morio* undergoes holometabolism, characterized by a life cycle comprising four stages: embryo, larva (caterpillar), pupa, and adult beetle. *Z. morio* larvae are used for feeding birds and reptiles (Rumbos and Athanassiou, 2021) and have even been considered nutrient sources for human consumption (Benzertiha et al., 2020; Scholliers et al., 2020). These larvae are readily available from commercial suppliers worldwide, including in the US and Europe. Unlike *G. mellonella*, *Z. morio* can be sustained at room temperature for extended periods and does not pupate under crowded conditions, simplifying long-term studies and transportation logistics. Maintaining *Z. morio* larvae within the temperature range of 25°C to 37°C facilitates their use in *in vivo* studies of host-pathogen interactions (Zou et al., 2022), and *Z. morio* larvae being larger than *G. mellonella* makes experimental manipulations easier. In a previous study, *Z. morio* was utilized in toxicology analyses of plant extracts (Zou et al., 2022), and in other studies, one of its close relatives, *Tenebrio molitor*, served as a model system for fungal infections (de Souza et al., 2015; Canteri de Souza et al., 2018). In 2023, the complete genome assembly of *Z. morio* became available (Kaur et al., 2023), sparking a significant surge of interest in this organism within the research community.

## Materials and methods

### Bacterial strains and preparation

G257, a carbapenem-resistant *A. baumannii* clinical isolate from our laboratory collection, was used in all studies. Two ATCC reference strains (*A. baumannii* ATCC19606 and *A. lwoffii* ATCC15309) were used as controls. In fitness assays, G422, a multidrug-resistant (MDR) carbapenem-resistant *A. baumannii* clinical isolate from our laboratory collection, was shown to have low fitness in a previous study (Nutman et al., 2020) (Table 1).

**Table 1:** General characteristics of the bacterial strains used in this study.

Strain	Relevant characteristic	Sample source	Fitness (CFU/thigh)	Reference or source
*A. baumannii ATCC19606*	Reference strain	ND	3.78	Sigma
*A. lwoffii ATCC15309*	Reference strain	ND	ND	Sigma
*A. baumannii G257*	Clinical strain, carbapenem-resistant	Bronchoalveolar Lavage	10.08	AIDA
*A. baumannii G422*	Clinical strain, carbapenem-resistant	Bronchoalveolar Lavage	5.23	AIDA

For various experiments, bacteria were cultured at 37°C on blood agar (tryptic soy agar supplemented with 5% sheep blood; Hylabs, Rehovot, Israel), MDR Acinetobacter agar (Hylabs, Rehovot, Israel), or brain–heart infusion (BHI) broth. Bacteria were grown at 37°C to the logarithmic phase (OD_600_ of 0.6). Cells were spun down, washed with phosphate-buffered saline (PBS), and then diluted to indicate cell density. The infecting inoculum was freshly prepared before the experiment and was confirmed by CFU quantification with serial dilutions.

### Invertebrate animal model

*Zophobas morio* was grown and maintained on sterilized sawdust (Meital Laboratories, Shekhanya, West Galilee, Israel). The larvae, in the final instar larval stage, were shipped and maintained in a plastic box with ventilation openings, at room temperature. Freshly washed vegetables were provided every 2 days for hydration. Larvae with an average weight of 640–800 mg were used within a month after arrival.

*G. mellonella* larvae in the 3-day instar larval stage (Biosystems Technology, Brierley Hill, UK) were stored at 25°C in a plastic box with ventilation openings. Larvae with an average weight of 200–300 mg were used within a week after arrival.

### *Zophobas morio* infection survival assay

Larvae were randomly divided into treatment groups of 30 larvae each. A 0.5 mL, 31 G syringe was used to inject 25 μL of aliquots of bacteria into the proleg of each larva. After injection, larvae were incubated in plastic containers at 25°C or at 37°C as specified, and the number of dead larvae was scored daily until the end of the experiment (days 5–7). Larvae were considered dead when they displayed no movement in response to touch.

### Bacterial fitness

Groups of 5 *G. mellonella* larvae were infected with 10^5^ CFU bacteria/larvae. Groups of 4 *Z. morio* larvae were infected with 10^7^ CFU/larva. Larvae were collected at 0 h and 4 h post-infection and homogenized individually in 1 mL of PBS using the gentleMACS Dissociator (Miltenyi Biotec, Bergisch Gladbach, Germany). Serial dilutions of homogenate in PBS were plated on MDR Acinetobacter plates and incubated at 37°C for 24 h, followed by CFU quantification. Non-manipulated larvae of each species (n = 4) and PBS-injected larvae of each species (n = 4) served as controls.

### Neutropenic murine thigh infection model

We tested *in vivo* growth using a neutropenic murine thigh infection model. Fitness was defined based on the CFU count 24 h after the injection of an inoculum of 10^5^ CFU. A detailed protocol was described previously (Nutman et al., 2020). The protocol was approved by the Institutional Animal Care and Use Committee at Tel-Aviv Sourasky Medical Center.

### Histopathology studies

Larvae were injected with PBS (control) or with 10^6^ CFU of G257 (infected). At time points of 0 and 16 h post-infection, larvae were washed with PBS, fixed with 70% ethanol, and stored at 4°C. Paraffin-embedded slides were prepared (Hepatho Lab Ltd., Rehovot, Israel), and H&E staining was performed as described (Barnoy et al., 2017).

### Antimicrobial efficacy

*Zophobas morio* larvae (30 per group) were injected into a proleg with 10^7^ CFU/larva of the bacterial strain. At the 30 min post-infection time point, a single dose of antibiotics, either 200 μg/mg ampicillin or 60 μg/mg meropenem, was injected into a different proleg. *Z. morio* larvae injected with PBS (30 per group) were used as controls. Survival and fitness assays were performed as described above. At least three independent experiments were performed with similar results.

### Statistical analysis and Bioinformatics

Statistical analysis was performed using GraphPad Prism 9 software (version 9.3.1, GraphPad Software Inc., San Diego, CA, United States). The Kaplan–Meier survival curves were generated. For two-group comparisons, a two-tailed unpaired t-test was applied. For multigroup comparisons, an analysis of variance (ANOVA) with Turkey–Kramer’s multiple-comparison test was used. A value of p of <0.05 was considered statistically significant. To build a phylogenetic tree, the cytochrome C amino acid sequences of each organism were retrieved from NCBI and aligned using MAFFT (Katoh et al., 2002). A phylogenetic tree was constructed using the NJ algorithm. Accession numbers were as follows: *Homo sapiens* AAP36314.1; *Mus musculus* NP_031834; *Gallus* NP_001072946.1; *Drosophila melanogaster* NP_001260509.1; *Danio rerio* NP_001002068.1; *Caenorhabditis elegans* P19974.2; *Rattus norvegicus* NP_036971.1; *Galleria mellonella* XP_052756993.1; *Zophobas morio* GCA_027724725.1 (Cytochrome C amino acid sequence was extracted from the full genome).

## Results

### Comparison of *Galleria mellonella and Zophobas morio*

**Figure 1:**
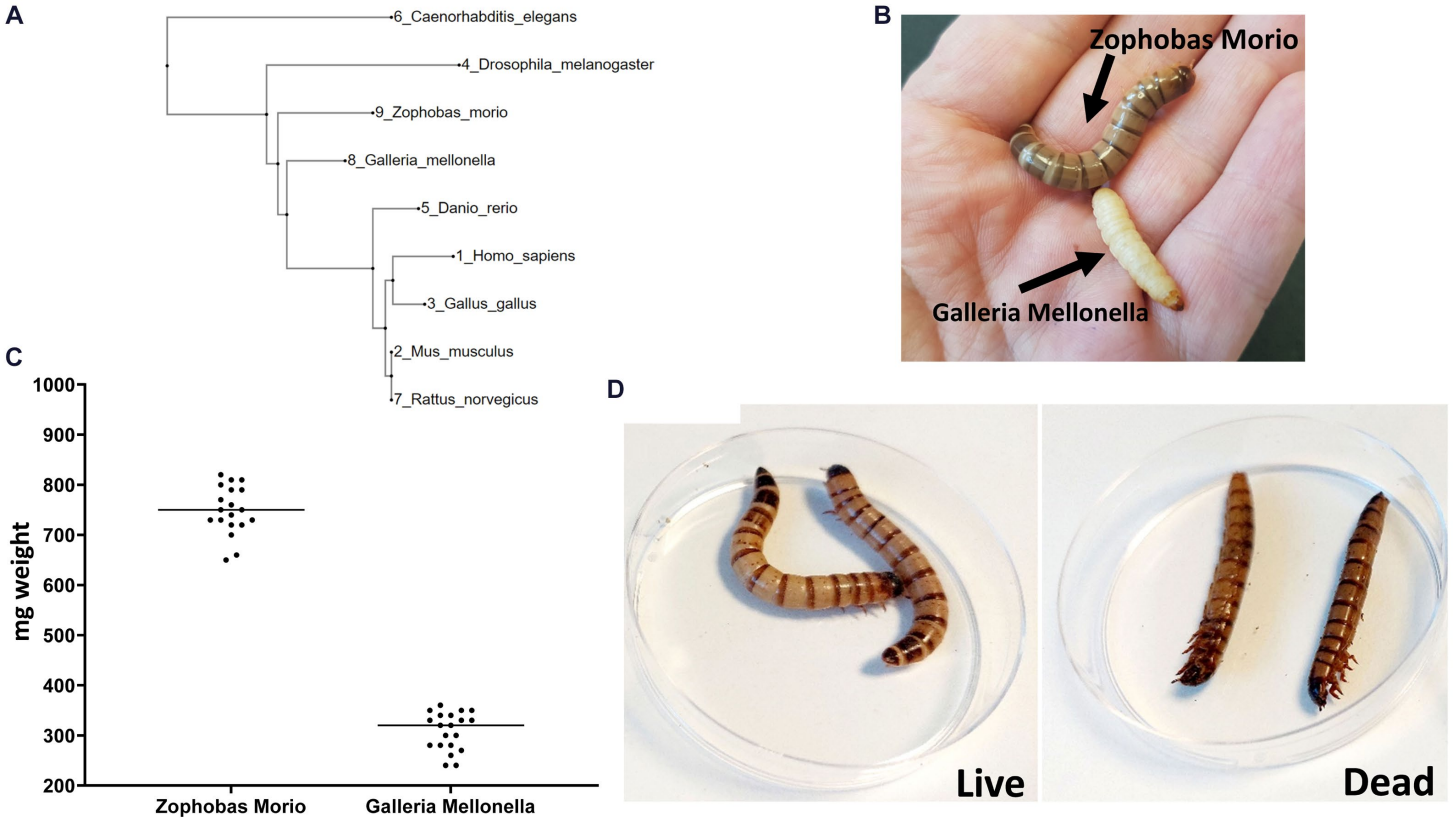
*Zophobas morio*. **(A)** A phylogenetic tree showing the most widely model organisms. **(B)** *G. mellonella* and *Z. morio*. **(C)** Weight range of *Z. morio* and *G. mellonella* larvae. Average weight is 758 mg per larva for *Zophobas morio* and 308.5 mg per larva for *Galleria mellonella*. **(D)** Live (left) and dead (right) phenotype of *Z. morio*.

We constructed a phylogenetic tree to measure the distance between *Z. morio* and other invertebrate and vertebrate animals used as infection models (Figure 1A). The phylogenetic distance from humans to both *Z. morio* and *G. mellonella* is comparable and shorter than the distance from *C. elegans* to humans. We then compared key phenotypic features of the *Z. morio* larvae to those of *G. mellonella*. *Z. morio* is larger and darker than *G. mellonella* (Figure 1B). The average weight of the *Z. morio* larvae was nearly 2.5-fold higher than the average weight of the *G. mellonella* (758 ± 14.2 mg vs. 308 ± 8.4 mg) (Figure 1C). The differences in size and weight allowed for easier handling and injection in the *Z. morio* model. Like *G. mellonella*, dead *Z. morio larvae* are easily distinguishable from live ones*—*they appear straight and rigid and do not respond to tactile stimuli (Figure 1D). The differences between the two models and their comparison to *C. elegance* are presented in Supplementary Table S1.

### Establishing *Zophobas morio Acinetobacter* spp. infection model

We used two laboratory reference strains as controls: a non-virulent *Acinetobacter lwoffii* ATCC15309 and *Acinetobacter baumannii* ATCC19606 (Table 1). As a clinically relevant strain, we used a previously described (Nutman et al., 2020; Frenk et al., 2022) *A. baumannii* G257 strain isolated from a patient with severe infection. This strain was previously found to harbor various virulence factors (Supplementary Table S2) and to be highly virulent in a mouse model, with a fitness of 10.08 CFU/thigh.

**Figure 2:**
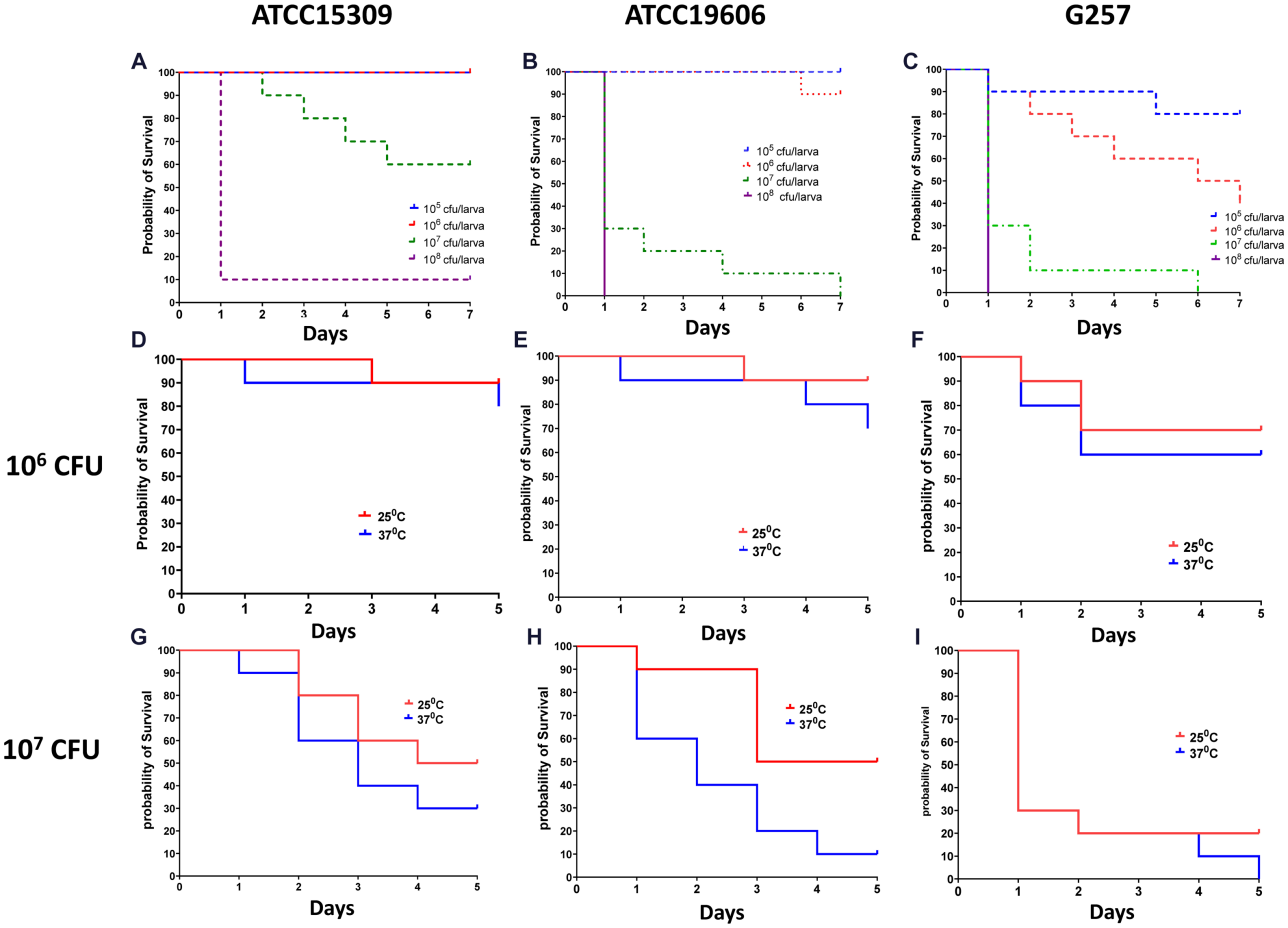
Dose and temperature effect on *Z. morio* larvae survival after infection with three different *Acinetobacter* strains. The Kaplan–Meier plot of *Z. morio* survival over 7 days. **(A–C)** Effect of inoculum dose on *Z. morio* survival during incubation at 37°C **(A)** *A. lwoffii* ATCC15309. **(B)** *A. baumannii* ATCC19606 **(C)** *A. baumannii* clinical isolate G257. **(D–F)** Effect of temperature on *Z. morio* survival. Panels **(D–F)** - inoculum of 10^6^ CFU/larvae; Panels **(G–I)** - inoculum of 10^7^ CFU/larvae. **(D,G)** - *A. lwoffii* ATCC15309. **(E,H)** - *A. baumannii* ATCC19606 **(F,I)** - *A. baumannii* clinical isolate G257. In each experiment, 20 larvae were injected with PBS as an injection control, with 100% survival in all cases.

We first established a survival assay. Infection was induced in *Z. morio* by injecting with the three study strains—*A. lwoffii* ATCC15309, *A. baumannii* ATCC19606, and the clinical *A. baumannii* isolate G257. Groups of 30 larvae were injected with 10^5^ CFU–10^8^ CFU of bacteria and incubated at 25°C or at 37°C for the indicated number of times. A group of 30 larvae injected with PBS and a group of 20 larvae without any manipulation were used as controls. Figure 2 shows the lethal dose effect of the three strains in a dose-dependent and temperature-dependent manner. A clear dose–response was observed for all three strains, as well as differences in the lethality between the strains (Figures 2A–C). At an inoculum of 10^5^ CFU/larvae, lethality was observed only among larvae infected with *A. baumannii* G257 (80% survival on days 5–7). At an inoculum of 10^6^ CFU/larvae, all larvae infected with *A. lwoffii* ATCC15309, 90% of those infected with *A. baumannii* ATCC19606, and only 40% of those infected with *A. baumannii* G257 survived at day 7. At an inoculum of 10^7^ CFU, higher lethality was observed in all three groups. The effect of temperature varied for different inoculums. At an inoculum of 10^6^ CFU, no significant differences in the survival of larvae at 25°C or 37°C were observed (value of *p* >0.05) (Figures 2D–F). At an inoculum of 10^7^ CFU/larvae, ATCC19606 was significantly more lethal at 37°C than at 25°C (*p* value <0.05) but not for the two other strains used (value of *p* = 0.29 for ATCC15309 and *p* value = 0.406 for G257) (Figures 2G–I).

**Figure 3:**
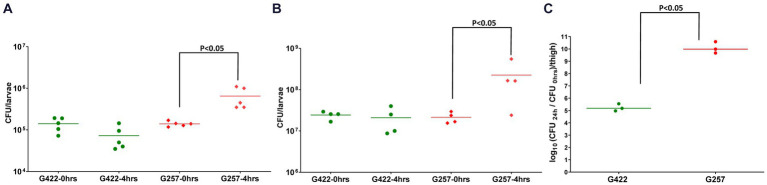
Fitness of two MDR *A. baumannii* clinical isolates in *G. mellonella* **(A)**, *Z. morio* **(B)**, and mice **(C)** *G. mellonella* larvae were injected with 10^5^ CFU/larva, and *Z. morio* were injected with 10^6^ CFU/larva, either with G422 isolate or with G257 isolate. Immediately (0 h) or 4 h after injection, larvae were ground and plated on MDR Acinetobacter Agar plates for CFU quantification. Untreated and PBS-injected larvae were sampled at both time points, and no colonies were detected on the selective plates. **(C)** Neutropenic mice (*n* = 3) were injected with 10^5^ CFU/thigh, either with G422 or with G257 isolate. Untreated and PBS-injected mice (*n* = 3 for each group) were used as control groups.

We next determined the *in vivo* fitness of *A. baumannii* in *Z. morio* by quantifying bacterial CFU 4 h after injection. As the larvae are not sterile, to specifically quantify the injected *A. baumannii*, we plated the bacteria on a selective medium (containing meropenem 4.5 mg/mL). As both reference strains used in this study are sensitive to meropenem, we used a clinical MDR isolate G422 as a control. G257 has high fitness in a mouse model (10.08 CFU/thigh), while G422 has lower fitness in a mouse model (5.23 CFU/thigh) (Figure 3C). The fitness of the two isolates in *Z. morio* larvae was compared with their fitness in *G. mellonella* larvae and was consistent with that of the two isolates in the mouse model (Figure 3). G422 was less fit, and both models did not show an *in vivo* increase in bacterial count. In contrast, strain G257 showed a significant increase in cell counts in both models (there was a 0.5-log increase in *G. mellonella* and a one-log increase in the *Z. morio* model, *p* < 0.05).

**Figure 4:**
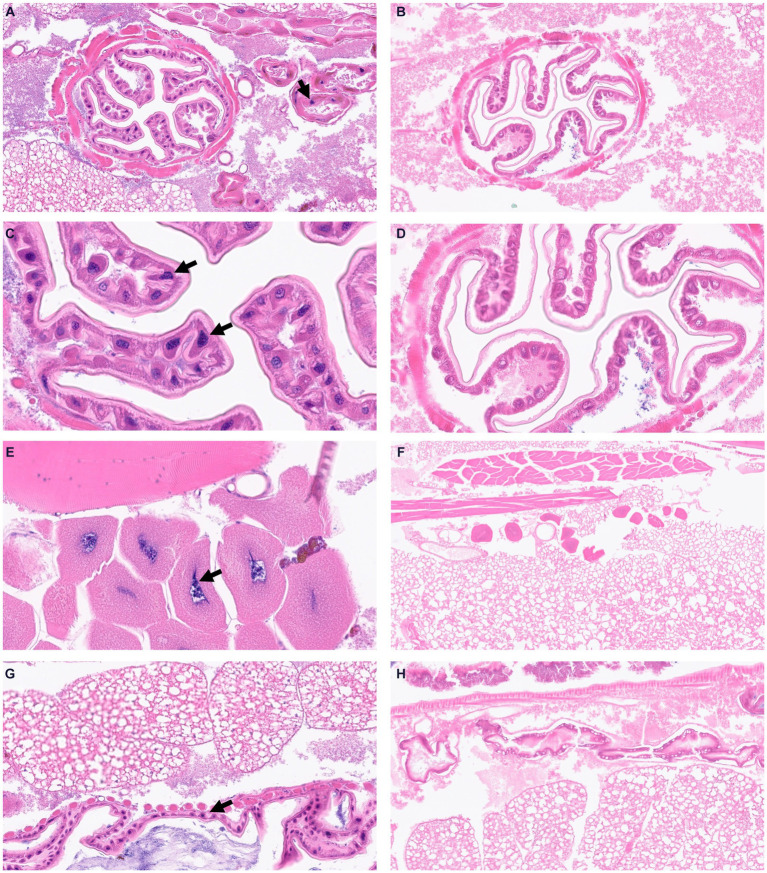
Visualization of *Z. morio* larvae tissues stained by hematoxylin and eosin. **(A–D)** Larvae were injected with G257 isolate at 1 × 10^7^ CFU/larvae and incubated at 37°C for 16 h. Black arrows indicated enlarged hemocytes containing bacteria. **(E–H)** Larvae were injected with PBS and incubated at 37°C for 16 h.

To visualize structural alterations, cellular abnormalities, or tissue damage caused by the infection, we visualized whole body sections of infected *Z. morio* larvae with H&E staining. At 16 h post-infection with the *A. baumannii* G257 strain, H&E stained cross-sections of larvae revealed clusters of hemocytes exhibiting phagocytosis of bacteria (blue dots throughout the section), with a small area of melanization around hemocyte aggregates (Figures 4A,B). Moreover, we observed hemocytes present in nodules, with some attached to organ structures with evidence of melanization (Figures 4C,D,G). In contrast, cross-sections of H&E-stained PBS-injected larva looked healthy (mock infection), with individually distributed hemocytes showing no noticeable aggregates of melanization (Figures 4E,F,H).

### Antimicrobial efficacy study

We used this novel infection model to test the efficacy of antimicrobials against *Acinetobacter* spp. We tested the efficacy of ampicillin and of meropenem against *A. baumannii* ATCC19606 (ampicillin-resistant and meropenem-susceptible, MIC = 1 mg/L for meropenem and MIC >32 mg/L for ampicillin) and against *A. baumannii* G257 (ampicillin and meropenem resistant, MIC =128 mg/L for meropenem and MIC >32 mg/L for ampicillin). We used a lethal inoculum (5×10^7^ CFU) and compared *Z. morio* survival after a single dose of ampicillin (200 mg/kg) or meropenem (60 mg/kg).

**Figure 5:**
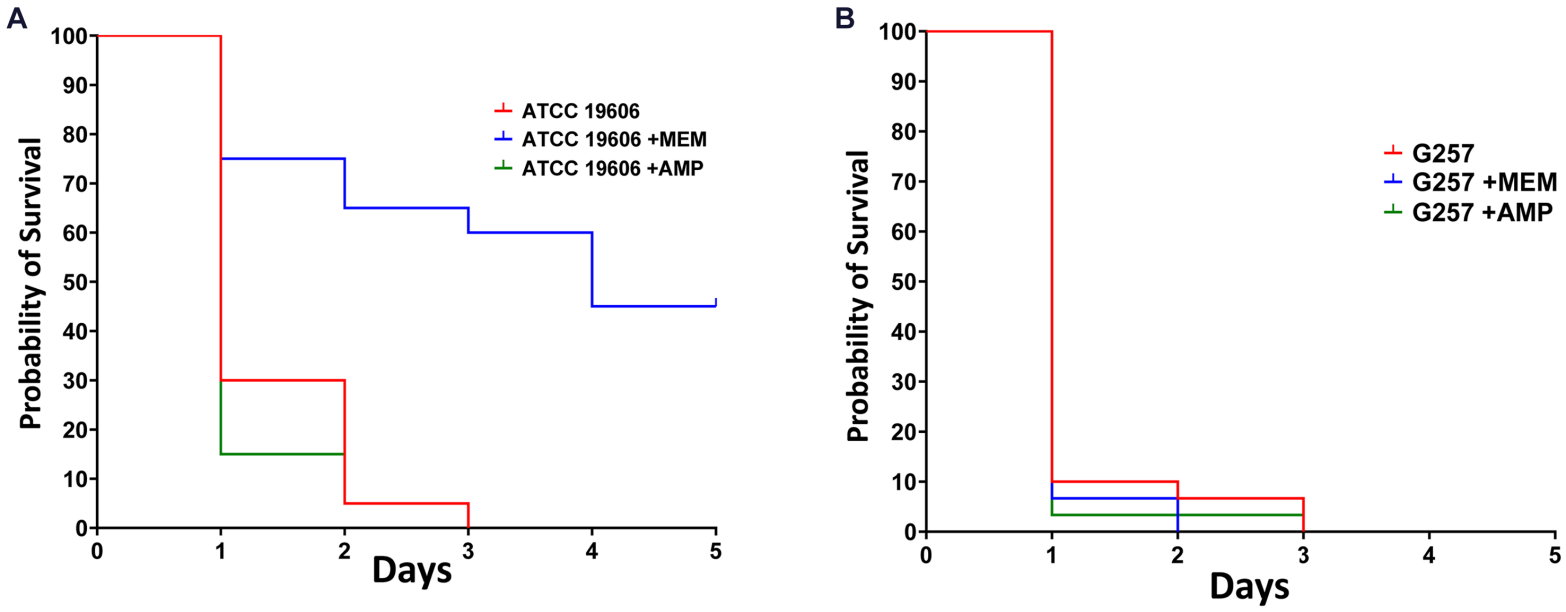
Meropenem that is active against *A. baumannii* ATCC19606 can prolong the survival of *A. baumannii*-infected *Z. morio* larvae. **(A)** After infection with 5×10^7^ CFU/larvae of *A. baumannii* strain ATCC19606, meropenem to which the strain was susceptible significantly prolonged the survival of *Z. morio* larvae compared with no antibiotic treatment (*p* < 0.001). However, ampicillin, to which the strain was resistant, caused no difference in survival compared with no antibiotic treatment. **(B)** After infection with a 5×10^7^ CFU/larvae of *A. baumannii* strain G257, meropenem, or ampicillin, to which the strain is resistant, there is no difference in survival compared with no antibiotic treatment.

By day 3, there were no surviving larvae of *Z. morio* infected with *A. baumannii* ATCC19606. Treatment with ampicillin, to which this strain is resistant, had no effect on larvae survival. On the other hand, treatment with meropenem, to which this strain is sensitive, significantly increased larvae survival (*p* < 0.001), with 45% surviving at day 5 (Figure 5A). All *Z. morio* infected with MDR *A. baumannii* G257 died by day 3, and treatment with either antibiotic had no effect on their survival (Figure 5B).

## Discussion

New invertebrate models are sought as an alternative to rodent studies. In this study, we present a novel infection model in the invertebrate *Zophobas morio*. We established the parameters of infection with *Acinetobacter* spp. H&E staining demonstrated *Z. morio’s* immune response to *A. baumannii* infection, which included hemocyte clusters engaging in bacterial phagocytosis, tissue melanization, and hemocyte aggregation. A similar immune response was reported by Peleg et al. in the *G. mellonella Acinetobacter* spp. infection model (Peleg et al., 2009). We showed an inoculum-dependent lethality of *Z. morio* after *Acinetobacter* infection. Survival depended on the virulence of the infecting strain, and we demonstrated that this infection model can be used to evaluate the *in vivo* fitness of the infecting strain. Meropenem treatment, but not ampicillin, resulted in improved survival of *Z. morio* larvae when the infecting strain was meropenem-susceptible but not if the strain was meropenem-resistant. This demonstrates that the model is suitable for testing the efficacy of antimicrobial therapy as well as the antibiotic resistance of bacterial strains.

*A. baumannii* is an important MDR nosocomial pathogen, for which very limited treatment options exist. Developing new antimicrobial agents that target *A. baumannii*, especially carbapenem-resistant strains, is of high priority. Invertebrate models may assist in screening and early testing of new agents. While *G. mellonella* is an important and established model to study *A. baumannii* infections, supply, rearing, and maintenance are challenging (Tao et al., 2021; ten et al., 2023). We believe that the *Z. morio* model is a valuable addition to the *in vivo* study of *A. baumannii* and potentially other bacterial pathogens.

The *Z. morio* infection model has various advantages over other invertebrate models. It is easy to obtain high-quality larvae, ship them, and maintain them. The large size allows easy manipulation during injections, and the results obtained using this model were consistent with those of other, more established model systems. While we found that the temperature of incubation of *Z. morio* larvae after infection might affect the results in some cases, we successfully used an incubation temperature of 37°C, compatible with optimal bacterial growth.

Further study is required to extend the use of this model to other pathogens and to examine the efficacy of additional antibacterial agents. In-depth examination of the immune response of *Z. morio* to infections, such as hemocyte response with fluorescently labeled *A. baumannii*, might provide further insights into the full potential of the model.

In conclusion, this study introduces a novel infection model for studying *A. baumannii* infection *in vivo*—in *Z. morio* larvae. The model can be used to study bacterial virulence and fitness and assess the efficacy of antimicrobial agents.

## Data availability statement

The original contributions presented in the study are included in the article/Supplementary material, further inquiries can be directed to the corresponding authors.

## Ethics statement

The animal study was approved by Sourasky Medical Center - Institutional Animal Care and Use Committee. The study was conducted in accordance with the local legislation and institutional requirements.

## Author contributions

NR: Conceptualization, Formal analysis, Investigation, Methodology, Writing – original draft, Writing – review & editing. ET: Writing – review & editing. AH: Methodology, Writing – review & editing. ML-W: Methodology, Writing – review & editing. AK-P: Validation, Writing – review & editing. YC: Project administration, Resources, Supervision, Writing – review & editing.
