# Supplementary material for: Zophobas morio larvae as a novel model for the study of Acinetobacter virulence and antimicrobial resistance
Source: Front Microbiol. 2024 Feb 27;15:1375787. doi: 10.3389/fmicb.2024.1375787 (PMC10927975; doi:10.3389/fmicb.2024.1375787)
Supplement: Supplementary file 1 [file Table_1.docx]

| **Supplementary Table 1**. Comparison of three invertebrate *in vivo* models. | | |  |  |  |  |
| --- | --- | --- | --- | --- | --- | --- |
|  |  |  |  |  |  |  |
|  |  | ***Z. morio*** |  | ***G. mellonella*** |  | ***C. elegans*** |
|  |  |  |  |  |  |  |
| Host Complexity |  | As complex as *G. mellonella* |  | More complex than *C. elegans* |  | Less complex |
|  |  |  |  |  |  |  |
| Lifespan |  | Long (can be keep at RT for 2-3 month) |  | Short (10 days) |  | Intermediate (2-3 weeks) |
|  |  |  |  |  |  |  |
| Temperature range |  | Grown at RT, can be kept at 37°C |  | Grown at 18°C, can be kept at 37°C |  | Grown and kept at RT |
|  |  |  |  |  |  |  |
| Immune response |  | Innate immune system, hemolymph with antibacterial properties, phagocytic hemocytes |  | Innate immune system, including phagocytic hemocytes |  | Conserved immune pathways, no adaptive immune system |
|  |  |  |  |  |  |  |
| Ease of genetic manipulation |  | No tools for manipulation |  | Limited tools for manipulation |  | Highly amenable to manipulation |
|  |  |  |  |  |  |  |
| Cost/availability |  | Low/high |  | High/low |  | Low/high |
|  |  |  |  |  |  |  |
| Phylogenetic relevance |  | As related to humans as *G. mellonella* |  | More related to humans than *C. elegans* |  | Distant phylogenetic relationship to humans and other mammals |
|  |  |  |  |  |  |  |
| Relevance to specific pathogens |  | Unknown |  | Used to study bacterial and fungal pathogens |  | Suitable for studying various pathogens, including bacteria, fungi and some viruses |
